# Brainjacking in deep brain stimulation and autonomy

**DOI:** 10.1007/s10676-018-9466-4

**Published:** 2018-07-30

**Authors:** Jonathan Pugh, Laurie Pycroft, Anders Sandberg, Tipu Aziz, Julian Savulescu

**Affiliations:** 1 The Oxford Uehiro Centre for Practical Ethics, University of Oxford, Oxford, UK; 2 Oxford Functional Neurosurgery, University of Oxford, Oxford, UK; 3 Future of Humanity Institute, University of Oxford, Oxford, UK

**Keywords:** Brainjacking, Deep brain stimulation, Autonomy, Security, Responsibility

## Abstract

'Brainjacking’ refers to the exercise of unauthorized control of another’s electronic brain implant. Whilst the possibility of hacking a Brain–Computer Interface (BCI) has already been proven in both experimental and real-life settings, there is reason to believe that it will soon be possible to interfere with the software settings of the Implanted Pulse Generators (IPGs) that play a central role in Deep Brain Stimulation (DBS) systems. Whilst brainjacking raises ethical concerns pertaining to privacy and physical or psychological harm, we claim that the possibility of brainjacking DBS raises particularly profound concerns about individual autonomy, since the possibility of hacking such devices raises the prospect of third parties exerting influence over the neural circuits underpinning the subject’s cognitive, emotional and motivational states. However, although it seems natural to assume that brainjacking represents a profound threat to individual autonomy, we suggest that the implications of brainjacking for individual autonomy are complicated by the fact that technologies targeted by brainjacking often serve to enhance certain aspects of the user’s autonomy. The difficulty of ascertaining the implications of brainjacking DBS for individual autonomy is exacerbated by the varied understandings of autonomy in the neuroethical and philosophical literature. In this paper, we seek to bring some conceptual clarity to this area by mapping out some of the prominent views concerning the different dimension of autonomous agency, and the implications of brainjacking DBS for each dimension. Drawing on three hypothetical case studies, we show that there could plausibly be some circumstances in which brainjacking could potentially be carried out in ways that could serve to enhance certain dimensions of the target’s autonomy. Our analysis raises further questions about the power, scope, and necessity of obtaining prior consent in seeking to protect patient autonomy when directly interfering with their neural states, in particular in the context of self-regulating closed-loop stimulation devices.

Technological developments in neuro-interventions have raised the prospect of ‘brainjacking’, that is, the unauthorized control of another’s electronic brain implant (Pycroft et al. 2016). It might seem somewhat natural to assume that the prospect of brainjacking represents a profound threat to individual autonomy. Whilst this is partly true, such a straightforward analysis obscures that fact that the prospect of brainjacking actually raises a number of complex questions regarding autonomy. In this paper, we attempt to elucidate the implications of this phenomenon by reflecting further on the nature of brainjacking in Deep Brain Stimulation (DBS), and the different dimensions of autonomy.

To frame our discussion in this paper, consider the following three hypothetical case studies in which examples of brainjacking plausibly raise very different questions about individual autonomy.

- Case One
  Alex suffers from Parkinson’s Disease and has validly consented to undergo DBS in order to ameliorate his motor impairment. The treatment is highly effective; however, a malevolent third party has gained control over Alex’s device, and is able to cease or change the parameters of his stimulation.
- Case Two
  Betty is a severely anorexic patient who has consented to undergoing DBS for anorexia nervosa. The treatment appears to have a positive effect on Betty’s eating behaviours. However, Betty becomes reluctant to continue with the stimulation because she embraces her anorexia as a part of her identity. Following this decision, her parents are becoming increasingly worried about her deteriorating condition. They begin to consider whether it might be possible to take control over Betty’s stimulation.
- Case Three
  Carl suffers from Parkinson’s Disease, and has validly consented to undergo DBS in order to ameliorate his motor impairment. Although the stimulation is successful in reducing his tremor, Carl is one of the rare patients who experiences off-target effects following stimulation. Whilst undergoing stimulation, he exhibits hypersexual behaviour that he does not exhibit in the absence of stimulation. Carl understands this, and chooses to stimulate himself only in the safety of his own home. However, a malevolent third party intentionally initiates stimulation whilst Carl is running errands in town, and Carl commits an act of sexual harassment. It quickly becomes clear to Carl’s treatment team and the police that Carl’s implant had been hacked at the time of his action. However, Carl does not feel substantially different now his stimulation has ceased. He begins to wonder whether he is his ‘right self’ now, and whether he can ever be regarded as responsible for his actions when there is always the possibility that his brain implant has been hacked.

Our aim in this paper is to detail how navigating the kind of concerns raised by these cases requires a nuanced understanding of different dimensions of autonomy. Rather than adopting a specific conception of autonomy and applying it to this context, our aim in this paper is to map out some of the conceptual territory of autonomy in order to fully elucidate the potential implications of brainjacking for different dimensions of autonomous agency. In doing so, we hope to make salient the need to develop adequate protections against brainjacking in DBS systems, but also to draw attention to circumstances in which benevolent brainjacking might potentially be understood as enhancing rather than undermining autonomy. We begin by providing some scientific background about brainjacking in DBS, before motivating this paper’s investigation by drawing on the burgeoning ethical literature discussing brainjacking and autonomy in the context of Brain Computer Interfaces. In section two, we provide an analysis of two different dimensions of autonomous agency before bringing this analysis to bear on the above case studies of brainjacking in section three.

## Scientific background

### Deep brain stimulation

DBS is a neurosurgical procedure in which electrodes are surgically implanted into precisely targeted areas of the patient’s brain, allowing physicians to alter neural function and thereby change behaviour. Since its widespread adoption, DBS has successfully been used in the treatment of tens of thousands of patients for neurological conditions such as Parkinson’s Disease (PD) and dystonia. It is also being considered as an experimental treatment for various other indications, including clinical depression (Delaloye and Holtzheimer 2014) and anorexia nervosa (Lipsman et al. 2013). DBS has also been proposed for the purposes of cognitive enhancement (Hu et al. 2009) and correcting abnormal moral behaviour (Fumagalli and Priori 2012). For example, Fuss et al. (2015) discuss the use of DBS as a treatment strategy to reduce sexual drive in paraphilic patients at high risk or re-offending.

The intended effects of DBS on both an individual’s physical abilities and mental life can be profound. When used as a licensed therapy for PD, the majority of patients experience positive outcomes from DBS, in terms of relief of disease symptoms and improved quality of life (Rodriguez-Oroz et al. 2005). However, like any medical treatment, DBS is not without side-effects and risks. In addition to the perioperative risks of the procedure itself, DBS has in some cases been observed to have various adverse cognitive, psychiatric, behavioural or psycho-social side-effects (Clausen 2010). Many of these side-effects and risks have received considerable ethical attention elsewhere (Baylis 2013; Clausen 2010; Gilbert 2013, 2017; Gilbert et al. 2017; Klaming and Haselager 2010; Kraemer 2013a; Lipsman and Glannon 2013; Schermer 2011), in light of the issues they raise for personal identity, responsibility, autonomy and well-being.

A DBS system consists of electrodes implanted in the brain using a stereotactic frame, connected via subdermal wires to an implantable pulse generator (IPG), which is typically implanted in the chest. The IPG sends electrical pulses to the brain, which alter the behaviour of neuronal tissue, resulting in changes in overall brain activity. This in turn can result in various effects ranging from the diminishment of motor impairment (in patients suffering from PD and other movement disorders), to altering the patient’s motivational or emotional states. The precise effect of the stimulation varies depending on the location of the electrodes and on the pattern of stimulation used, as determined by the surgical team based on the patient’s diagnosed condition and on a range of physiological indicators. Different electrode contacts can be selected, enabling stimulation of slightly different regions, and stimulation parameters such as amplitude, pulse width, and frequency can all be varied.

Control over these parameters is affected by alteration of the software settings of the IPG. The IPG contains a battery, microprocessor, memory, and radiofrequency antenna. This enables it to receive communications from an external “programmer”—a device that can be used by patients and clinicians to program parameters into the IPG wirelessly. Clinicians are able to set the overall parameters so that the stimulation is optimally effective—each patient has a slightly different optimum, so personalisation is necessary for good clinical outcomes. Patients can then use a less complex programmer to change settings within a pre-defined limit established by the clinician.

### Neurological implant cyber-security

Based on developments in information security research, there is reason to believe that it would be technically feasible for a third party to interfere with the software settings of an IPG without the patient’s or clinician’s consent; a process referred to as “brainjacking” (Pycroft et al. 2016). Such an attack on a DBS system is, to the best of our knowledge, currently hypothetical. However, documented attacks on other implanted medical devices (such as insulin pumps and cardiac pacemakers), and Brain Computer Interfaces suggest that the risk of such an attack occurring may not remain merely hypothetical for long (Pycroft et al. 2016) .

The exertion of such control over an IPG could allow a hacker to directly influence the patient’s brain function, thereby affecting their behaviour. Interfering with IPG settings would enable the hacker to alter a patient’s brain activity in a manner determined by the site of stimulation, the patient’s pathological condition, their neuroanatomical configuration, and the design of the IPG. Simple instantiations of these attacks wherein the hacker does not require detailed knowledge of the patient’s condition—“blind attacks” (Pycroft et al. 2016)—may include attempts to damage brain tissue by over-stimulation and denial of treatment by switching off the stimulator.

More complex attacks wherein the hacker utilises patient-specific knowledge of the DBS system being attacked—“targeted attacks”—would enable more intricate influence to be exerted over the patient (Pycroft et al. 2016). Such potential attacks could include exacerbation of symptoms—impairing PD patients’ movements, or causing chronic pain patients additional pain; induction of impulse control disorders, which may substantially impair patients’ ability to control socially unacceptable behaviour; alteration of emotional affect; and potentially use of reinforcement learning to “train” patients to engage in (or refrain from) certain behaviours, as directed by the hacker. We shall elaborate on some of these potential mechanisms in greater detail below.

In either blind or targeted attacks, the recipient of DBS no longer exerts control over their stimulation. Little attention has been given to the ethical implications of this in the neuroethics literature. Two notable exceptions are Ienca and Andorno’s (2017) discussion of putative human rights in this context, and more saliently for our purposes Ienca and Haselager’s (2016) ethical exploration of brainjacking in the context of Brain–Computer-Interfaces (BCIs). In their discussion, although Ienca and Haselager identify DBS as a neuro-stimulator that may be particularly vulnerable to brainjacking, they instead focus on brainjacking BCIs, since the possibility of hacking these devices has been proven in both experimental and real-life settings. Ienca and Haselager are correct to note that this is not true of brainjacking in DBS. However, we believe that the possibility of such attacks is sufficiently plausible to make ethical reflection appropriate.

We shall conclude this section by briefly surveying some of the ethical concerns relating to brainjacking in DBS that significantly overlap with the ethical concerns pertaining to brainjacking in BCIs that Ienca and Haselager (2016) raise. First, the possibility of brainjacking represents a potential risk of both kinds of intervention, and such risks may need to be disclosed to patients in order to enable adequately informed consent, particularly if the feasibility of brainjacking increases. Second, brainjacking in both contexts raises privacy concerns. In the case of DBS, blind attacks may gain access to sensitive information stored on the IPG including the patient’s name, diagnosis, stimulation parameters, and their physician’s details (Pycroft et al. 2016). That said, it is unlikely that brainjacking of currently employed DBS systems^1^ could be used to extract the wide array of information that could be extracted by brainjacking a BCI device, such as financial information (Martinovic et al. 2012). Third, both kinds of brainjacking could be used to exert physical and psychological harm. In the case of BCIs, the harm exerted by hacking would consist in taking away the benefit of the BCI in assisting the user’s physical and psychological performance (Ienca and Haselager 2016). Whilst an analogous kind of harm is possible in the case of brainjacking DBS, attacks in this latter context could also induce further significant harms. For instance, third party alteration of stimulation frequency could induce severe pain in the recipient, whilst alteration of the pulse width could induce unpleasant off-target effects, and could even induce brain tissue damage (Pycroft et al. 2016).

Whilst the above ethical concerns regarding brainjacking are significant, and although there are some slight differences in the specifics of how the issues are raised in the context of DBS and BCIs, Ienca and Haselager’s discussion of these issues in the latter context translates quite straightforwardly to the former. As such, we shall set these concerns to one side and instead focus on the implications of brainjacking in DBS for individual autonomy. We take this to be the most significant ethical issue raised by brainjacking generally, and it is an issue that Ienca and Haselager (2016) leave unresolved in their discussion. They note:

…although hacked BCI-users with severe neurological conditions would be exposed to the risk of diminished autonomy if compared to non-hacked users with the same condition, they may, nevertheless, achieve greater overall autonomy than equally impaired patients who do not have access to BCI whatsoever. This fact is worth extensive philosophical reflection, since the counterintuitive situation that the same technology can both increase and diminish autonomy requires quite detailed analysis of the benefit-risk ratios in different scenarios (Ienca and Haselager 2016).

We join Ienca and Haselager in rejecting the simplistic claim that brainjacking undermines autonomy. Our aim in the remainder of this paper is to provide the sort of extensive philosophical reflection that these authors call for on different understandings of autonomy. To be clear, we do not intend to advocate one single approach to autonomy as the *correct* approach; rather, our intention here is to highlight some key approaches to different dimensions of autonomy, and how they might be brought to bear on the cases that we outlined at the outset of this essay.

### Different dimensions of autonomous agency

Concerns about autonomy raised by brainjacking are particularly salient because of the significant value that we attribute to autonomy in contemporary bioethics. As well as being understood to bear significant prudential value (Young 1982), philosophers in the Kantian tradition have commonly understood autonomy to undergird our high moral status. Autonomy is also typically understood to be related to a number of other important values including personal identity, authenticity, agency, and responsibility.

However, there is also significant disagreement about the nature of autonomy; indeed, the counter-intuitive situation that Ienca and Haselager refer to in the above quotation is, in large part, a reflection of the various ways in which we can interpret the concept of autonomy. We shall take as our starting point Dan Brock’s broad definition of autonomy, according to which autonomy.

…involves the capacities of individuals to form, revise over time, and pursue a plan of life, or conception of their good. It is a broad concept, applicable at both the levels of decision and of action. (Brock 1993).

Of course, further meat needs to be added to the bones of this broad definition. First, it seems clear that an adequate theory of autonomy should place certain conditions upon the sorts of desires or ‘plans of life’ that we can appropriately be said to hold autonomously. After all, we can sometimes fail to be autonomous even when we act in accordance with some of our desires. For instance, Harry Frankfurt offers the illuminating example of an unwilling drug addict as one illustration of this phenomenon (Frankfurt 1971). The addict who feels compelled to take a drug may plausibly be said to ‘desire’ the drug at a first order level; however, we may doubt that the addict is autonomous with respect to his decision to take the drug, if she nonetheless repudiates that desire at a higher order level, and wishes that it didn’t move her to action.

One particularly influential approach to responding to such cases has been to appeal to conditions pertaining to the ‘authenticity’ of the agent’s motivating desires; on accounts that endorse such conditions, a motivating desire can only form the basis of an autonomous decision if the agent herself somehow identifies with that desire. For our purposes here, it will be useful to attend to a distinction between what Alfred Mele (1995) terms “internalist” and “externalist” accounts of this aspect of autonomy. According to internalist accounts, the fact that some desire satisfies a certain sort of internal psychological scrutiny by the agent is both necessary and sufficient for establishing that the desire is authentic. For example, classic internalist theories have suggest that, in order to be autonomous with respect to my desire to engage in some behaviour X, I must reflect on whether I want this desire to X to be effective in moving me to act (Frankfurt 1971), or perhaps on whether I think that I have a reason to X (Watson 1975). In contrast, externalist accounts deny that this psychological scrutiny is sufficient; such accounts claim that a necessary condition of autonomy is that the *causal history* of the desire that grounds a particular decision must meet certain conditions (Christman 1991; Mele 1995).

Charitable externalists may allow that internalist theories identify some necessary (though not sufficient) conditions of autonomy. However, some externalists deny even this to internalist theories. Notably, in outlining their influential account of autonomy in bioethics, Tom Beauchamp and James Childress deny that autonomous decision-making must be grounded by authentic desires in the internalist sense; they believe that this would make autonomy too difficult to achieve (Beauchamp and Childress 2009). However, they agree with the externalist that autonomous decisions must be made in a particular sort of way; a decision will only qualify as autonomous if it is made (i) intentionally, (ii) with substantial understanding, and (iii) in the absence of controlling influences that determine action, such as coercion, manipulation and deception (Beauchamp and Childress 2009).

The accounts briefly sketched above pertain to a dimension of autonomy that has also been understood to be a central feature of the concept of moral responsibility. Indeed, many theorists seem to treat the concept of moral responsibility as being almost co-extensive with the concept of autonomy, at least as the latter is understood to pertain to some sort of critical reflection on one’s own motivational states or their causal history. For example, in their recent discussion of the implications of DBS for moral responsibility, Sharp and Wasserman appeal extensively to John Christman’s diachronic account of autonomy (Sharp and Wasserman 2016). Conversely, in their discussion of the autonomy and authenticity of enhanced personality traits, Bublitz and Merkel (2009) understand autonomy as “an agent’s status of being an apt target for reactive attitudes such as praise and punishment”. Such a definition, appealing as it does to Strawsonian reactive attitudes, is suggestive of an intimate connection between autonomy and moral responsibility.

This sort of intimate relationship between autonomy and responsibility can also be seen elsewhere in the broader philosophical literature on the concepts of moral responsibility and autonomy. This is perhaps partly a result of the fact that both concepts have historical roots in the long-standing free will debate, and the importance of voluntariness to each concept. 2 Although we believe that important distinctions can be drawn between the concepts of autonomy and moral responsibility, we do not have space to address this complex issue here. Rather, we shall follow others in treating the two as co-extensive. However, we take this to be justified in the current context, since in this paper we are particularly interested in the conditions of control that have understood to be central to both autonomy and responsibility across the two literatures.^3^ The degree of control required for moral responsibility, is, we believe, also necessary for what we shall term decisional autonomy (a term we shall introduce below), although it may not be sufficient; autonomy is thus, on the conception that we employ here, conceptually narrower than responsibility.

Let us return to Brock’s definition of autonomy, which we outlined at the beginning of this section. Recall that this definition states that the autonomous agent must not only be able to form and revise plans—they must also be able to *pursue* them. For Brock then, autonomy incorporates a *practical* dimension. Autonomy is not just about how agents make their decisions; it is also about whether they are in a position to act in their pursuit. This approach to the nature of autonomy is perhaps quite natural in the practical contexts in which autonomy is frequently discussed; indeed, when medical ethicists claim that personal autonomy has particularly salient value, and that doctors have a duty to respect a patient’s treatment decisions, they often seem to implicitly assume that autonomy also incorporates this sort of practical dimension. Nonetheless, the suggestion that autonomy incorporates this practical dimension has also proven contentious. For instance, it has also been claimed that extending the concept of autonomy in this way is to confuse autonomy with liberty (Coggon and Miola 2011), or even the nature of autonomy with its value (Taylor 2009).

As such, it seems that there are three closely related, and perhaps even overlapping concepts at work in this area: autonomy, moral responsibility, and liberty. We cannot enter into the debate about precisely how we should philosophically distinguish these concepts here. Rather, in the interests of clarity, we shall employ the following somewhat stipulative definitions, which we believe broadly maps onto the way in which these concepts have been discussed in the literature: We shall use the term ‘decisional autonomy’ when considering questions pertaining to whether individuals are making their decisions in the light of their own desires and plans. Decisional autonomy as we understand it admits of the internalist and externalist accounts outlined above.

**Fig. 1:**
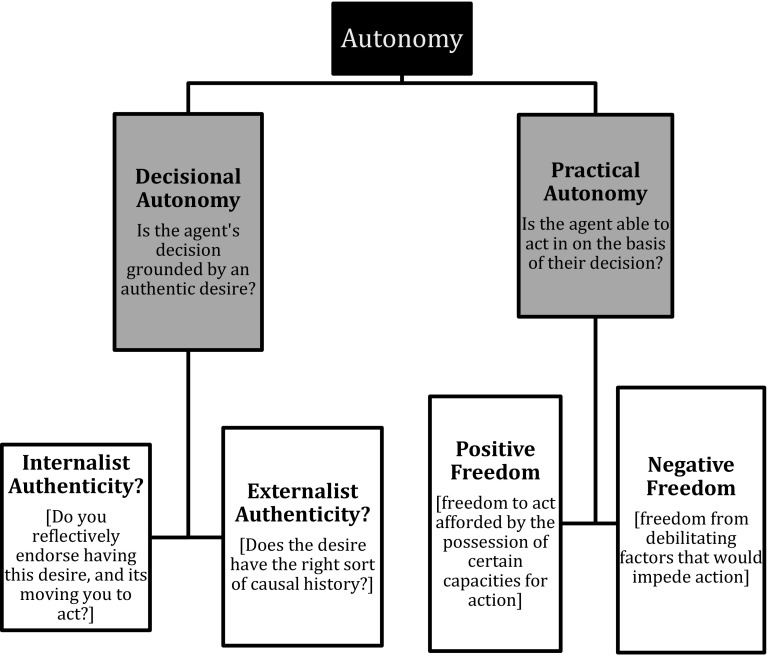
Breaking down different dimensions of autonomy

We shall use the terminology of ‘practical autonomy’ to refer to the agent’s ability to pursue these plans. In turn, practical autonomy can be understood to incorporate considerations of both negative liberty (that is, freedom from debilitating factors that would otherwise impede one’s action) and positive liberty (that is, the freedom afforded to one by the possession of certain capacities for action) (Berofsky 1995).^4^ We shall use the terminology of autonomy as an umbrella term in contexts where we mean to invoke considerations of both decisional and practical autonomy. This breakdown of the different dimensions of autonomy is illustrated in Fig. 1.

Prior to outlining the implications that DBS may have for these different elements of autonomy, and the attendant issues that brainjacking raises, we shall conclude this section by highlighting two further distinctions that are apposite here. The first is between *local* and *global* understandings of autonomy (Dworkin 1988). In a local sense, we may be concerned with whether an individual is autonomous with respect to a particular decision to act in some way in a specific instance, and with whether they have the necessary practical autonomy to act effectively on the basis of it. In contrast, in a global sense, we may be concerned with whether an individual is able to pursue certain ends (that they have made an autonomous decision to pursue) over the course of extended periods of time.

**Fig. 2:**
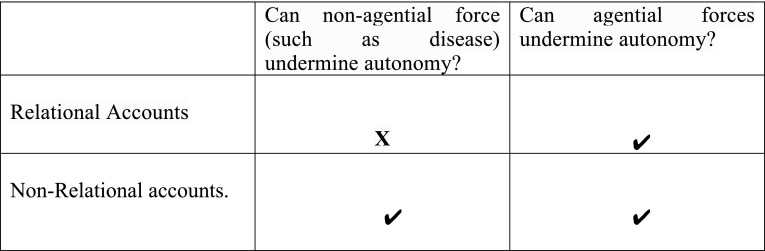
Relational accounts vs non-relational accounts

Second, the broad definition of autonomy that we have outlined here claims that the autonomous agent must base their decision on desires that are authentic in either an internalist or externalist sense. Notice that this definition leaves open the possibility that the authenticity of one’s desires may be undermined by *both* third party intentional interference and non-agential interference. However, on what we may term purely ‘relational accounts’ of decisional autonomy, this dimension of autonomy can *only* be undermined by third party agents (Bublitz and Merkel 2009; Taylor 2009). Such accounts have followed a recent trend in the philosophical literature that has sought to prioritise relational aspects of autonomy (Anderson and Honneth 2005; Mackenzie and Stoljar 1999; Westlund 2009).^5^ We shall call theories that allow for non-agential forces (such as disease) to undermine decisional autonomy ‘non-relational accounts’. Figure 2 highlights the differences between these two kinds of understandings of autonomy.

## Brainjacking and the different dimensions of autonomy

### Practical autonomy

Although brainjacking might potentially threaten the target’s practical autonomy, the sorts of threat posed by brainjacking to this aspect of autonomy are not particularly novel, as we shall explain. Accordingly, our analysis in this section shall be somewhat brief.

To see why the issues raised by brainjacking for practical autonomy are not particularly novel, consider first the point that the intended therapeutic end of any medical intervention is often the enhancement of the practical dimension of an agent’s autonomy. In the current context for instance, patients such as Alex in case one may undergo DBS to alleviate severe motor symptoms associated with PD. Such symptoms often prevent patients from actively pursuing various activities that may previously have been central to their conception of the good life. In so far as stimulation serves to alleviate these impediments to the patient’s acting on the basis of their desires to pursue these goods, stimulation can be understood to enhance their practical autonomy.

As such, non-consensual third-party control of a DBS could feasibly infringe upon an individual’s practical autonomy by ceasing the individual’s stimulation without their consent. This would serve to re-instate impediments to the individual’s practical autonomy. For instance, a targeted attack of a patient undergoing DBS for PD could plausibly impair the patient’s motor function by changing stimulation parameters (Pycroft et al. 2016).

However, the third party *initiation* of stimulation that serves to alleviate an impairment (or the third-party deployment of a BCI application) could also be detrimental for the agent’s practical autonomy all things considered. Notice that this would be true even if the stimulation nonetheless enhances the individual’s positive freedom, by virtue of enhancing their physical capacities. To see why, suppose that an individual has refused to consent to an instance of stimulation that would alleviate her motor impairment; if so, then initiating stimulation in spite of this (let us presume valid) refusal would still infringe upon the individual’s practical autonomy in an important sense; it would frustrate her desire not to be stimulated in that instance. Compare this to a case in which a patient rejects analgesics because she would prefer to be lucid and suffering, rather than pain-free but delirious. In so far as the individuals in both cases have validly refused these interventions, they have made the decision that not undergoing the intervention is more important to them than any potential positive effects that the intervention might have on her ability to act in accordance with other desires. The principle of respect for autonomy thus requires that the interventions are not carried be out in each case.

In such cases, it is crucial to distinguish the individual’s freedom to not have their stated preference frustrated, and the increase in freedom that non-consensual stimulation might afford. We should not assume that non-consensually increasing an agent’s physical capacities will enhance their practical autonomy all things considered just because it will enhance their capacity to act in certain ways—doing so will fail to respect autonomy if the agent herself prefers the absence of stimulation to the increase in physical capacity that stimulation affords. Indeed, Carl in case three has very good reasons to prefer not to undergo stimulation in certain contexts. Even though it may enhance his physical capacities by removing his motor impairment, it may give rise to impulsive desires that he strongly does not want to act upon.

What about instances in which the individual has neither consented to nor refused stimulation, perhaps because they have not been asked to consent? Here third parties face epistemic barriers to knowing whether initiating stimulation would frustrate the recipient’s preferences. Whether or not third party stimulation should be construed as enhancing the individual’s practical autonomy in this case would depend on the evaluative weight the recipient ascribes to the potential positive effects that stimulation might have on their ability to act in accordance with some desires, in comparison to the evaluative weight they might ascribe to not undergoing stimulation.

With respect to practical autonomy then, brainjacking may pose a significant potential threat to patients such as Alex in case one: although the attack may not directly affect Alex’s decisional autonomy, the unauthorized cessation of stimulation would rob Alex of the positive liberty afforded to him by stimulation. Conversely, the unauthorized initiation of stimulation might frustrate Alex’s preference not to undergo stimulation, even if it enhances his positive liberty more generally by ameliorating his motor impairment.

However, it is important to note that these sorts of threat are not restricted to brainjacking attacks. Rather, it seems that the hacking of any other medical devices could plausibly be construed as undermining practical autonomy in the ways described above. Hacking a pace-maker can have significant effects on one’s practical autonomy, in much the same way that hacking a DBS system can.^6^

### Decisional autonomy

Brian-jacking thus raises familiar issues with regards to the practical dimension of autonomy. However, it raises more complex issues about decisional autonomy. The reason for this is that it raises the prospect of a third-party holding direct control over the very processes that undergird our status as autonomous decision-makers. Hackers could plausibly exert direct influence over an individual’s deliberations by brainjacking DBS via a number of different mechanisms, as we shall explore in this section.

Of course, the possibility of taking advantage of these mechanisms depends on the victim having electrodes implanted in the appropriate area. However, with that caveat in mind, it is possible to identify at least three possible mechanisms that might be exploited by brainjackers. First, stimulation of the subthalaminc nucleus (STN) in patients suffering from PD assists in the management of impulse control disorders (ICDs) that are relatively common amongst such patients. By disrupting stimulation parameters in this context, hackers could remove the protection that DBS affords against such ICDs, or even induce aberrant impulse control (Pycroft et al. 2016). Second, hackers could stimulate inappropriate electrode contacts in order to induce personally and socially undesirable emotional changes. Alteration of emotional processing is often an unintended side-effect of STN- DBS in the treatment of PD (and stimulation of the Nucleus Accumbens for other indications). However, some uses of DBS in the psychiatric context have deliberately targeted areas associated with emotional processing in order to modulate the dysregulated affective states that characterise certain psychiatric disorders (Lipsman and Lozano 2014). Third, a number of emerging DBS indications in psychiatry target neural circuits associated with reward processing, such as the Nucleus Accumbens; with a sufficient degree of control of the IPG, a hacker could plausibly initiate stimulation to reinforce certain behaviours (such as eating behaviours in the case of an anorexic patient) as a form of operant conditioning to modify the behaviour of the victim (Pycroft et al. 2016).

Existing discussions of the implications of DBS for decisional autonomy have focused on whether recipients of *consensual* DBS can be autonomous with respect to actions undertaken as a result of the *unintended* side effects of consensual stimulation (Klaming and Haselager 2010; Kraemer 2013b; Maslen et al. 2015). The focus on consensual DBS is not surprising; due in large part to the highly invasive nature of the procedure required to implant the physical components of a DBS system, and its experimental nature for certain indications, there is a broad consensus that valid consent should be obtained prior to DBS treatment (Nuttin et al. 2014). Moreover, as various authors have pointed out, individuals who undergo consensual DBS sometimes report experiences of self-estrangement or alienation following stimulation, in part due to the development of novel psychological characteristics. In turn, it has been argued that such estrangement could plausibly serve to undermine personal autonomy (and/or moral responsibility).

The phenomenon of self-estrangement following consensual DBS, and its implications for autonomy (and/or moral responsibility) has received a great deal of attention in the neuroethics literature that we lack the space to fully address here (Baylis 2013; Clausen 2010; Gilbert 2013, 2017; Gilbert et al. 2017; Klaming and Haselager 2010; Kraemer 2013a; Lipsman and Glannon 2013; Schermer 2011). However, in discussing brainjacking, we are considering an unprecedented potential avenue for the non-consensual stimulation of patients who have previously provided valid consent to the implantation of physical components of a DBS system. This, we believe raises a new set of questions for the autonomy (and/or moral responsibility) of recipients of DBS. Whilst these questions are our primary focus in this section, the existing prior discussions of the implications of unintended side-effects of consensual DBS for decisional autonomy provide a useful background for understanding the implications of brainjacking DBS for autonomy.

Sharp and Wasserman (2016) have recently addressed the implications of unintended side-effects of consensual DBS for decisional autonomy, although, as we mentioned above, they phrase their discussion in terms of moral responsibility rather than autonomy. According to this account, an agent is only autonomous with respect to an action if it is issued from a psychological characteristic that she would not be alienated from following hypothetical reflection (unconstrained by distorting factors) on the historical processes that gave rise to it (Sharp and Wasserman 2016). Crucially, Sharp and Wasserman also incorporate a notion of tracing into this historical account, according to which our assessment of responsibility for some act at *t* + *1* can *trace back* to an action at *t* for which the agent was responsible. So, although a drunk driver may not exert control over their driving behaviour whilst drunk, we can hold them responsible for their driving in so far as we can trace back their responsibility for their choice to drink prior to driving (Sharp and Wasserman 2016). Similarly, in the context of DBS, an individual can be held responsible for impulsive behaviour under stimulation if he does not feel alienated from the historical processes that gave rise to his initial decision to undergo stimulation, and if he foresees the likely effects of stimulation on his behaviour.

Although the unintended side-effects of consensual DBS may plausibly undermine decisional autonomy, particularly on externalist accounts, we should note that there will likely be some cases in which the intended therapeutic effects of the consensual procedure may appropriately be construed as *facilitating* decisional autonomy. This will be so if stimulation can be construed as enhancing the agent’s competence, or their ability to critically reflect on their motivating desires (or their aetiology). As some of us have argued elsewhere, one scenario in which DBS might do this is if stimulation would serve to increase the individual’s ability to exert top–down control over competing compulsive or impulsive desires, or by reducing the motivational force of such desires, so that they do not move the agent to act prior to carrying out critical reflection about what to do (Maslen et al. 2015). On internalist accounts, stimulation might also arguably enhance decisional autonomy by serving to amplify the motivational force of desires that the agent herself endorses, but which lack sufficient motivational force to move her to act in the absence of stimulation. Alternatively, stimulation might remove other impediments to the sort of critical reflection that autonomy requires, such as pain.

Assessments of the effect of consensual DBS on autonomy are more complicated in cases in which stimulation has unintended deleterious effects on the competencies required for decisional autonomy, whilst simultaneously facilitating the patient’s ability to act in certain ways. In a much-discussed case described by Leentjens et al. DBS was able to effectively alleviate a patient’s severe motor incapacitation, but also led to stimulation-related mania that was not responsive to treatment. Whilst the patient was competent, he was asked to choose between continuing stimulation and being committed to a psychiatric ward, or remaining bed-ridden for the rest of his life due to his motor incapacitation. The patient chose stimulation (Leentjens et al. 2004).^7^

This case highlights the importance of the distinction between local and global understandings of autonomy, introduced in section II. With the terminology of this distinction in mind, we might understand the patient in Leentjen’s case to have made a locally autonomous decision to sacrifice his competence to make future locally autonomous decisions, by choosing to continue undergoing stimulation that renders him decisionally incompetent. Nonetheless, we may still understand his choice here as facilitating his global autonomy, in so far as stimulation allows him to achieve the end over time that he himself believes he has most reason to achieve, namely, not remaining bed-ridden due to his severe motor incapacitation. This choice can thus be construed as a kind of Ulysses contract (Unterrainer and Oduncu 2015); just as Ulysses tied himself to the mast in order to hear the Sirens’ song without swimming to his death, so might this patient have decided to live in a state of mania in order to be able to live the remainder of his life in a physically active manner. In both cases, the chosen impairment can be understood to enhance autonomy in an important sense, in so far as it is necessary for the agent to effectively pursue the goal that they have decided is most important *to them*, even if the impairment takes away the liberty to pursue other competing goals. In some cases of deciding whether or not to use a medical technology, it is not simply a case of choosing whether or not we want to increase the number of options available to us; sometimes, the relevant choice is between different kinds of mutually exclusive option sets.

As the above discussion makes clear, ascertaining the effects of even consensual stimulation on these different dimensions of autonomy raises complex questions. In some ways, third party interference via brainjacking may seem to make the issue simpler; if brainjacking means that a competent individual does not undergo stimulation voluntarily, it is difficult to see how they could be autonomous with respect to the actions that they performed as a result of that stimulation.^8^ Furthermore, it seems clear that third parties can seriously infringe upon another’s autonomy, either by ceasing stimulation that maintains an individual’s competence (so that the individual is no longer able to carry out the sort of critical reflection that decisional autonomy requires), or by ceasing stimulation that maintains an individual’s capacity to act, as we suggested above. In fact, brainjacking seems to be a paradigm case of the sorts of manipulative interference with autonomous decision-making that externalists in the autonomy literature have been at pains to highlight.

However, there are some less clear cases; for instance, it is less clear what we should say about cases in which a third party initiates non-consensual stimulation in order to *increase* the agent’s competence to make autonomous decisions. For instance, consider case two above; it might be claimed that Betty’s desire to refuse further stimulation evidences a lack of decision-making competence, in so far as her decision seems to be grounded by pathological values (Tan et al. 2007; Geppert 2015). On this approach, it might be claimed that we should respect her treatment decision when she is undergoing stimulation, in so far as stimulation serves to correct the aberrant neurological processes that underlie her pathological values without stimulation. Moreover, according to internalist accounts of decisional autonomy, if Betty, following non-consensual stimulation, decides to act in accordance with a desire to eat that she endorses following the appropriate sort of critical reflection, she is, by virtue of that fact, autonomous with respect to that behaviour.

However, as Sharp and Wasserman (2016) make clear, externalist accounts might plausibly object that third-party stimulation undermines decisional autonomy *even if* it increases the agent’s competence. It would do so if the individual herself would hypothetically take herself to have reasons to object to the causal history of a psychological characteristic that was efficacious in moving them to act. For instance, in case two, Betty might take herself to have reasons to object to the causal history of her post-stimulation desire to consume food, even though that stimulation might be understood as enhancing her decision making competence, at least on some understandings of that latter concept. Furthermore, on such externalist accounts, Betty could potentially qualify as autonomous with respect to her (non-stimulated) decision to refrain from eating if she embraces the ‘pathological’ process by which she developed this plan in a minimally rational sense (Christman 1991).

There are other circumstances in which it might plausibly be claimed that third parties may be in a better position than the agent herself to readily identify instances in which stimulation might be appropriate. Consider, for example the potential use of DBS to combat paraphilias in convicted sex offenders (McMillan forthcoming); even if stimulation could be effective in reducing sexual urges, such a device would fail to achieve the aim of reducing the targeted behaviour if the recipient were unable or unwilling to reliably initiate stimulation before his urges take hold. Criminal justice authorities might welcome the possibility of hacking such an offender’s device in the interests of public safety. Third party control here would be broadly analogous to closed-loop DBS systems in which the device itself interprets signals from sensory electrodes in the brain in order to determine when stimulation is required, as well as delivering stimulation (Wu et al. 2015). The benefit of third party control is that it would allow non-physiological predictors to be taken into account when deciding when stimulation might be necessary.

What are the implications of third party control for autonomy here? It seems that much will depend on the precise effects of stimulation, how the recipient views his sexual urges, and the basis of the recipient’s prior consent to initially having the physical components of the DBS device implanted. If the recipient authorized third party control over his device, then autonomy-based concerns significantly reduce; however, such authorized third party control would not accurately be construed as ‘brainjacking’.^9^

Suppose though that the agent consented to having the device implanted on the basis that he alone would be in charge of his stimulation. Here, concerns relating to the agent’s global autonomy become far more salient, even though the agent’s capacity to make locally autonomous decisions might plausibly be enhanced by stimulation. To illustrate, if stimulation served to increase the agent’s top–down control over an uncontrollable impulsive behaviour from which they feel alienated, then the third party initiation of stimulation could serve to increase his capacity to make locally autonomous decisions with respect to his behaviour on either internalist or externalist accounts of decisional autonomy. Nonetheless, if the agent did not feel alienated from their impulsive behaviour, and their lack of control over their urges, such stimulation would globally frustrate the agent’s practical autonomy to act in accordance with these urges. In such circumstances, an all things considered autonomy-based justification of brainjacking would not be applicable.

As such, the implications of brainjacking for decisional autonomy will depend not only on the particular features of the target’s situation, but also on the particular account of decisional autonomy that we endorse. For instance, in the case of Betty, the key to interpreting the case lies in the assessment of whether Betty is autonomous with respect to her decision under stimulation, or in the absence of stimulation. As we have described above, depending on the theory of decisional autonomy invoked, non-consensual stimulation could be construed as enhancing Betty’s decisional autonomy; however, if we maintain that we should respect Betty’s treatment wishes when she is off stimulation, non-consensual stimulation would amount to a serious violation of her negative freedom to act in accordance with this preference.

Carl’s situation in case three arguably raises a further set of relevant considerations here. It might be claimed that the internalist and externalist accounts we have been focusing on overlook the crucial relational aspect of why we might plausibly have autonomy-based concerns about third-party control over stimulation devices. Such concerns, it might be claimed, are best captured by relational understandings of autonomy, rather than non-relational accounts, as we shall now explain.

As we discussed at the end of section II, relational views make the strong claim that only intentional third party interference can undermine autonomy. Bublitz and Merkel put the point as follows:

Persons can be manipulated through various means, from the presentation of false evidence, hypnosis and advertisements, through to pharmaceutical interventions. It is not the means that render them nonautonomous but the fact that someone else *illegitimately infringed upon their rights*. (Bublitz and Merkel 2009)

Such accounts arguably lead to some implausible outcomes, as Sharp and Wasserman (2016) point out. For instance, it seems highly plausible that an individual may come to lack autonomy in the absence of a third party infringing their rights in some way. For example, those suffering from paraphilias may plausibly claim that they are not autonomous with respect to their behaviour because they are moved to act by irresistible impulsive desires that they repudiate at a reflective level. They seem analogous to Frankfurt’s unwilling addict, described in section I.

Rather than adopting a strong relational view, it seems more plausible to adopt a compromise position that rejects the strong relational claim, but that also affords some significance to third party interference. Such a compromise position might claim that although third-party interference is not necessary for undermining autonomy all things considered, it is necessary to undermine a particular kind of freedom incorporated into our overall conception of agency, namely freedom from domination.^10^ The thought here is that in so far as a third party is able to exert control over the individual’s stimulation, the third party is able to exert significant power over that individual.

This sense of freedom has its roots in the republican tradition of political liberty.^11^ A salient aspect of this conception of freedom for our purposes is that the violation of this freedom does not require actual interference with one’s liberty; it can be violated simply by the fact that one is in a situation in which another could hypothetically exert arbitrary power over another.^12^ To illustrate, we may say a slave lacks freedom from domination, even if he is subject to a benign slave-owner who never tells him what to do, but who could exert this power if he so chose. This is relevant to the present discussion because those who stress the importance of this freedom might plausibly claim that the *mere possibility* of a third party hacking a DBS device renders individuals with those devices lacking this sort of freedom. On this sort of account, the need to develop sophisticated forms of cyber-security to protect stimulation devices becomes all the more salient.

Indeed, this is the sort of situation in which Carl in case three seems to find himself in. The damage of the hack is not just that it leads Carl to commit an act of sexual harassment; it robs him of his ability to understand himself as an autonomous agent. Whilst a degree of scepticism about one’s ability to act and decide in a perfectly autonomously manner is a good thing, complete scepticism can in fact undermine autonomy. These reflections raise the daunting prospect that if brainjacking became frequent and difficult to detect, individuals with DBS devices could no longer guarantee that their behaviour had been autonomous, since any action of theirs could feasibly have been influenced by (undetectable) hacking. Not only would this undermine claims of responsibility after a wrongful action has been undertaken, it would also undermine the agent’s ability to take responsibility for their own future actions. This would amount to clinical teams being put into the curious, self-defeating position in which they may be motivated try an improve a patient’s autonomy by implanting a device that *de facto* casts doubts on the recipient’s autonomy.

## Conclusion

We should not be surprised by the supposedly counter-intuitive notion that hackable neurotechnologies such as BCIs and DBS may both advance and diminish autonomy. In this paper we have drawn attention to cases in which brainjacking raises difficult conceptual questions about individual autonomy. The analysis that we have provided here helps to illuminate not only how brainjacking can have a variety of effects on different aspects of autonomous agency, but also how different understandings of the dimensions on autonomous agency can have significant implications for our understanding of the threat that brainjacking may (or may not) pose to individual autonomy. As our analysis of the above case studies suggests, the implications of brainjacking for autonomy are complex, and will depend significantly not only on the nature of the third party interference in question, but also on which element of autonomous agency the hack effects, and how that particular element functions in one’s overall understanding of the nature and value of autonomy. In so far as brainjacking can positively affect one dimensions of autonomy whilst negatively affecting others, further work is required to develop a theory of the value of these different dimensions of autonomy.

Although we have phrased our discussion in the language of autonomy rather than moral responsibility, our analysis here may be taken to have important implications for moral responsibility. The precise extent of these implications of course depends on how one conceives of the relationship between autonomy and moral responsibility. Whilst we have not attempted to fully address this complex question here, we have suggested that the degree of control required for moral responsibility is also necessary for decisional autonomy, although it may not be sufficient. Accordingly, on the understanding that we have sketched here, the mere fact that brainjacking can serve to undermine decisional autonomy in the ways described above does not alone entail that it would also undermine the agent’s moral responsibility. In order to establish that brainjacking undermines moral responsibility, we would need to provide a more detailed account of the control conditions of moral responsibility. Although we have not provided such an account here, and acknowledging the possibility that there is an important conceptual space between autonomy and moral responsibility, it seems plausible that brainjacking could in some circumstances undermine an agent’s control to a degree that is sufficient to undermine their moral responsibility. Just as an individual who is involuntarily intoxicated may not be morally responsible for their actions whilst intoxicated, so too may an individual who has been subjected to  brainjacking lack moral responsibility for actions performed under stimulation, if stimulation can plausibly be understood to have significantly affected the agent’s decision-making.^13^ Conversely though, brainjacking might also be used to enhance the control competencies required for morally responsible behaviour.

In any case, we hope to have outlined some different conceptual tools that will be of use in navigating questions pertaining to the autonomy of subjects in the sort of morally complex cases of brainjacking we have discussed here. Moreover, we should not lose sight of the fact that there are likely to be far more straightforward cases of brainjacking that do pose significant threats to autonomy. In view of the salience attributed to autonomy in contemporary bioethics, developers (and clinical teams) have a moral duty to further consider the security of neural implants currently in use, and if possible to develop greater protections against brainjacking.

Finally, our discussion of autonomy in this context raises further questions for the ethics of self-regulating closed-loop stimulation devices. If we are concerned about the absence of patient control in brainjacking, this raises the question of whether similar concerns would be raised by the consensual use of closed-loop systems, which employ algorithmic decision-making to determine when stimulation is carried out (Gibert 2017; Goering et al. 2017; Kellmeyer et al. 2016). Both brainjacking and closed-loop systems can serve to take the patient ‘out of the decision-making loop’—the ethical question is whether consensually substituting the patient’s judgment with an algorithm in a closed loop device is always more respectful of their autonomy than non-consensually substituting the patient’s judgement for another benevolent, intentional agent in brainjacking. More fundamentally, these issues raise questions about the power, scope, and indeed necessity of obtaining prior consent in seeking to protect patient autonomy when directly interfering with their neural states.

## Footnotes

1 However, it should be acknowledged that it may be possible to gain access to further information from closed loop DBS systems, which combine the recording features of a BCI with the stimulation of conventional DBS.

2 See Fischer (1999), Mckenna (2002).

3 Theories of moral responsibility often incorporate epistemic conditions pertaining to the foreseeability of certain outcomes. For a discussion of the implications of DBS for this condition of responsibility, see Klaming and Haselager (2010).

4 Some have questioned the utility of the distinction between positive and negative liberty (Feinberg 1984; MacCallum 2006). However, this has no bearing on the issues we discuss here. Even if one cannot draw a useful distinction between these aspects of freedom, that does not mean that the freedoms under consideration are not relevant to practical autonomy.

5 For a discussion of relational identity in the context of DBS, see Baylis (2013).

6 This is not to say that interventions with these devices are morally equivalent in all respects. It might be argued that the embodied nature of brain implants makes it more likely that individuals will incorporate their implant into their self-conception than external devices. Moreover, given the broad understanding of the role of the brain in facilitating consciousness and undergirding our individual characteristics, we may be particularly concerned about the effect that brainjacking will have on the victim’s self-conception in comparison to the hacking of other internal medical devices (such as a pace-maker). We do not wish to advance this claim here; our concern at this point is only on the implication that brainjacking has for practical autonomy.

7 See Glannon (2010) for further discussion.

8 See Klaming and Haselager (2010) for a discussion of attributing moral responsibility in such cases.

9 A further complicating factor for the validity of consent in this context arises if the recipient only consents to having the physical components of the DBS device implanted as a condition of his parole, or in return for a reduced sentence. As a number of commentators have argued in the literature on chemical castration, such an offer might arguably introduce coercive pressure that threatens the validity of the recipient’s consent. See Green (1986), Vanderzyl (1994). For further discussion of coercion in this context, see Bomann-Larsen (2013), McMillan (2014), Pugh (forthcoming), Wertheimer and Miller (2014).

10 Wertheimer comes close to adopting this sort of view when he distinguishes a narrow conception of voluntariness according to which voluntariness is only undermined by external constraints, from a broader conception, according to which voluntariness can be undermined by internal constraints. See Wertheimer (2012).

11 See, for instance, (Costa 2009; Pettit 1997; Skinner 1998).

12 Ibid; (Pettit 1996).

13 However, for a discussion of the conceptual difficulty of ascertaining whether changed capacities or behavioural patterns are attributable to DBS, see Gilbert et al. (2017), Gilbert (2017).
